# Managing diabetes and hypertension in western Kenya: A qualitative study of experiences of patients supported by the primary health integrated care for chronic conditions (PIC4C) model of care

**DOI:** 10.1371/journal.pgph.0003245

**Published:** 2024-08-15

**Authors:** Violet Naanyu, Ruth Willis, Jemima Kamano, Hillary Koros, Adrianna Murphy, Pablo Perel, Ellen Nolte

**Affiliations:** 1 School of Arts and Social Sciences, Moi University, Eldoret, Kenya; 2 Academic Model Providing Access to Health Care, Eldoret, Kenya; 3 Department of Health Services Research and Policy, Faculty of Public Health and Policy, London School of Hygiene and Tropical Medicine, London, United Kingdom; 4 Centre for Global Chronic Conditions, London School of Hygiene and Tropical Medicine, London, United Kingdom; 5 School of Medicine, Moi University College of Health Sciences, Eldoret, Kenya; 6 Department of Non-Communicable Disease Epidemiology, Faculty of Epidemiology and Population Health, London School of Hygiene and Tropical Medicine, London, United Kingdom; University of the Witwatersrand, SOUTH AFRICA

## Abstract

The Primary Health Integrated Care for Chronic Conditions (PIC4C) pilot project was launched in 2018 to strengthen prevention and control of four non-communicable conditions at primary health care level in western Kenya. We conducted a qualitative study to explore the extent to which PIC4C integrated services supported people with hypertension and/or diabetes towards timely diagnosis and referral, treatment, follow-up and adherence, from the perspective of those receiving care. Semi-structured interviews were conducted with a purposively sampled patient cohort at two time points, with the intention of capturing changes over time (total (n) = 43, completion of both interviews (n) = 37). We extracted existing survey data to describe socio-demographic characteristics and analyzed qualitative data thematically. We identified two cross-cutting contextual factors, individual’s financial resources and their social situation, which shaped each stage of their interactions with PIC4C services. The PIC4C model successfully engaged people in accessing screening services to enable timely diagnosis and referred them to enter care. Free community level screening services and decentralization of care to lower level facilities reduced cost barriers for patients. However, retention in care and adherence to treatment were affected by the wider system context in which PIC4C was operating, including inconsistencies in medication availability and patients’ limited financial capacity. Individually tailored advice from health care workers to work around some of these challenges supported self-management strategies. Further development of the service should focus on supporting health care workers to adopt flexible, contextually responsive approaches in order to support patients facing economic and other constraints to engage in (self) care.

## Introduction

The rising burden of non-communicable diseases (NCDs) in Kenya is creating a ‘triple’ disease burden of communicable disease, injuries and NCDs, with NCDs causing some 40% of deaths in 2019 and this share is expected to rise to over half by 2030 [1]. This poses considerable challenges to the health system, where lack of systematic screening and early detection of NCDs, as well as low capacity at primary level, lead to late diagnosis and advanced disease that require specialist input. This places increasing pressure on already stretched hospitals and their staff [1].

Strengthening primary health care (PHC) is widely recognised to be key to addressing the NCD burden [2] and the Kenyan government has committed to an integrated PHC approach [3] that makes NCD services “available, accessible, and affordable at all levels of care from the community level” [1, p. 26]. As part of this commitment, the government has supported the piloting of the Primary Health Integrated Care Project for Chronic Conditions (PIC4C) in western Kenya. Launched in 2018 for three years, PIC4C aimed to strengthen the prevention and control of hypertension, diabetes, breast and cervical cancer at primary health level [4]. It involved multiple components, including community health volunteer-delivered education, screening and referral; infrastructural measures such as equipment and electronic health records as well as strengthening the supply chain system using a revolving drug fund pharmacy model; structured referral pathways, decision-support tools and treatment protocols across the different tiers of the delivery system; and regular monitoring and evaluation [5].

The PIC4C model was evaluated using a process evaluation [6] and an implementation research project to inform and support scale up of integrated care for people with NCDs in Kenya [5]. Here we report findings of a qualitative study of patient experiences of PIC4C implementation conducted within the latter. Specifically, we explored the extent to which PIC4C integrated services supported people with hypertension and/or diabetes towards timely diagnosis and referral, and follow-up with and adherence to treatment, from the perspective of those receiving care.

## Methods

We report study methods in accordance with the standards for reporting qualitative research (SRQR) guidelines (S1 Checklist) [7] and the STROBE guidelines for reporting observational studies (S2 Checklist) [8].

### Study design

Our principal approach was semi-structured interviews with people with hypertension and/or diabetes. We explored their experience of services across the different levels of service delivery at two time points over the study period. This qualitative study was nested within a larger quantitative survey [9], and we extracted survey data to describe socio-demographic characteristics of the qualitative study participants.

### Study setting

The study was set in the mostly rural Busia and Trans Nzoia counties in western Kenya, with populations of between 900,000 and 1 million and high levels of poverty [5]. Each county has one county referral hospital (level 5), five (Trans Nzoia) to six (Busia) sub-county hospitals (level 4), seven (Trans Nzoia) to 12 (Busia) health centres (level 3) and between 38 and 47 dispensaries (level 2) serving up to 200 community units. PIC4C was implemented across 40 level 2–5 health facilities in Busia and 33 in Trans Nzoia.

### Sampling strategy and recruitment

Study participants were identified from respondents to a survey of patients’ experiences of treatment burden (n = 301) (12/2020-2/2021) who had indicated willingness to be contacted to participate in an interview. Details of the survey methods are reported elsewhere [9]. In brief, survey respondents had been screened and hypertension and/or diabetes confirmed through PIC4C service efforts between 2018 and 2020. For this qualitative study, we used purposive sampling, seeking to recruit 30–40 participants across the two counties, include a range of perspectives of those experiencing care stratified by gender, health condition(s), age and facility level, and allow for some anticipated loss to follow-up over interviews. We reviewed the diversity of patients in the sample and the data generated periodically during the first round of interviews, concluding that a final sample size of 40 was appropriate as all identified categories of experience were included, and no new substantive themes were being identified.

Potential participants were contacted by telephone (May–July 2021 and February–March 2022) by trained bilingual research assistants who explained the purpose of the interview and what participation would involve. For those agreeing to take part, an interview date most convenient for the participant was set. Participants were provided with an information sheet, which was read out to those who were unable to read and time given for questions. Informed consent was sought, and was recorded in writing and verbally at the beginning of the interview recording.

### Data collection

Interviews were held in person at a health facility closest to participants’ homes between 24 May and 8 July 2021 (round 1) and between 24 February and 4 March 2022 (round 2). This enabled exploration of patients’ experiences at different points in their trajectory since diagnosis and the setting-up and embedding of PIC4C services. Interviews were conducted by trained research assistants using a semi-structured interview guide, informed by literature on treatment and self-management burden [10–12], while also exploring people’s experiences of the health service (S1 File). The topic guide was adapted for the second interview to focus on changes in participants’ circumstances, and experiences of care and interactions with the health system since their first interview. We also explored patients’ recommendations for strengthening services, and for health workers introducing similar services in other counties. Interview guides were developed in English, translated into Swahili, piloted with four participants, reviewed by the field research team, adapted and finalised. Interviews were conducted in Swahili or English, audio recorded, transcribed and translated into English. Transcriptions and translations were reviewed for accuracy, and linked and cross-checked with survey data. Identifiable information (individual and health facility names, locations) was replaced with anonymous identifiers during transcription.

### Analysis

Demographic characteristics of the sample were described using existing survey data [9]. Interview transcripts were analysed using a thematic approach. This included familiarisation of data through reading and rereading transcripts, organising data according to pre-defined and newly identified topics, and synthesising and comparing data to identify core themes [13, 14]. We used NVivo 12 software to support qualitative data analysis [15]. We shared findings from a preliminary analysis of round 1 interviews in a stakeholder workshop in May 2022 with representatives from patient groups, community health workers and health facility staff, regional decision makers and the national Ministry of Health; feedback regarding areas to explore in greater depth informed subsequent analysis.

### Ethics review

The study received approvals from Moi University Institutional Research and Ethics Committee (FAN:0003586) and the London School of Hygiene & Tropical Medicine (17940), and a research permit from the National Commission for Science, Technology and Innovation (NACOSTI/P/20/4880).

### Inclusivity in global research

Additional information regarding the ethical, cultural, and scientific considerations specific to inclusivity in global research is included in the Supporting Information (S3 Checklist)

## Results

### Participant characteristics

We interviewed 43 patients, of whom 37 participated in both rounds; three participated in round 1 only (S1 Table) and three in round 2 only. Ten participants (23%) reported having diabetes, 19 (44%) hypertension and 14 (33%) both diabetes and hypertension (Table 1). Over half of participants (n = 27) were diagnosed within two years preceding the interview. Twenty-three were female and 19 male. The majority (n = 25) were educated to primary level. Income was low, with half of participants (n = 21) reporting average monthly household income of <3,000 KES (USD27 in January 2021 (1 USD/110 KES)) [16] during the previous 12 months. One quarter (n = 11) reported coverage by the National Health Insurance Fund (NHIF).

**Table 1:** Demographic and health characteristics of study participants.

	Busia County	Trans Nzoia County	Total
**Total Participants (n)**	20	23	43
**Reported condition(s) (n)**			
Diabetes	5	5	10
Hypertension	8	11	19
Diabetes & Hypertension	7	7	14
**Sex (n)**			
Female	12	11	23
Male	8	11	19
Data unavailable	0	1	1
**Age**			
Reported age range (years)	37–71	34–66	34–71
Mean reported age (years)	54.3	51.4	52.8
Data unavailable (n)	0	1	1
**Marital Status (n)** *			
Single/Separated/Widowed	4	3	7
Married	16	18	34
Data unavailable	0	2	2
**Educational attainment (n)** *			
No formal education	4	0	4
Primary education	14	11	25
Secondary education	2	8	10
Tertiary education	0	1	1
Data unavailable	0	3	3
**Monthly household income (Kenyan Shilling, KES), past year (n)** *			
<1000	2	2	4
1001–2999	9	8	17
3000–4999	5	3	8
5000–7999	2	3	5
8000–10000	0	1	1
10000–15000 **KSh**	0	1	1
>15000	2	2	4
Data unavailable	0	3	3
**National Health Insurance Fund (NHIF) coverage (n)** *			
Has current NHIF coverage	5	6	11
Does not have current NHIF coverage	15	15	30
Data unavailable	0	2	2
**Time since diagnosis (n)**			
<1 year	6	1	7
1–2 years	9	11	20
3–5 years	3	9	12
6–10 years	1	1	2
>10 years	1	0	1
Data unavailable	0	1	1

* Source: survey data collected December 2020-February 2021

### Cross-cutting contextual factors

We report findings by each stage of patients’ interactions with PIC4C integrated services from screening and diagnosis to referral and follow-up and ongoing treatment. We identified two inter-related contextual factors that dominated participants’ accounts across their diagnosis and treatment journey. These were: (i) individual financial resources and (ii) participants’ social situation. We first introduce these contextual factors, which informed interpretation of subsequent findings.

#### Financial resources: Heterogeneous economic situations and engagement in care

While all interview participants were seeking subsidised or free of charge care at public facilities, their individual economic circumstances varied. As noted above, about half of study participants had a monthly household income that was substantially below the rural poverty line in Kenya (KSh 3,947 per individual in 2021 [17]). We did not ask participants directly about their occupation or financial situation during interviews, but they raised how their economic circumstances affected their capacity to engage in care, for example starting descriptions of their experience of diagnosis or treatment by explaining that that they could not afford to seek care ‘*we did not have any income*’ [ID24; diabetic and hypertensive].

For many of our study participants, affording both basic daily living costs, and costs associated with health care, remained a struggle (‘*Sometimes you might not have even a single shilling*’ [ID05; diabetic and hypertensive]), and this shaped individuals’ experiences of receiving care through PIC4C services, with those who had fewer resources particularly valuing free services and encountering greater challenges with treatment costs.

#### Individual social situation

A second, inter-related contextual factor was the individual social situation of the participant. Some were embedded in family and social networks, and these networks were a source of support for some, helping them to access food or medicines to support treatment, for example:

*When they discovered I have the disease of sugar [diabetes], they became friends even more than before….When I need that clinic money, I can ask all of them and none will deny me….as long as they have it.* [ID30; diabetic]

For others, family responsibilities such as raising dependent children or grandchildren affected their prioritisation of resources and ability to engage in care provided by PIC4C:

*The challenges we encounter* [with managing diabetes and hypertension] *is sometimes when you lack money, sometimes your family also depends on you…. When I am overwhelmed…[…]… I have nothing to support them with* [ID07; diabetic and hypertensive]

There was a strong sense among some of needing to support others (‘*for us single mothers it is hard*. *Everything depends on you…*’ [ID26; diabetic and hypertensive]), highlighting the importance of social networks to enable them engaging with care:

*I stay alone with my kids, my husband left me with them….I keep thinking of how I could raise school fees for the kids and how they were supposed to have their daily meals since money was a problem. My children are the ones who started some hustles to enable me [to] sustain going for check-ups*. [ID09; diabetic]

The following sections report findings by each stage of patients’ interactions with PIC4C integrated services.

### Screening and diagnosis

Sixteen participants (37%) reported having initiated their patient journey at community level, generally through local screening events delivered by community health volunteers (CHV) linked to PIC4C. Prompts for attending screening varied. Some reported having experienced unexplained symptoms and seeing local screening events as an opportunity to seek health advice, while others responded to community health worker invitations to attend events. These events helped people who could not easily afford to pay for services to be diagnosed (*‘let’s go and be tested*, *it is free’* [ID42; hypertensive]), reducing the need to travel as screening services were *‘brought to the villages’* and enabling patients to be ‘*found*’ by community health workers:

*If it weren’t for them [CHVs], I would still be chilling out there, and perhaps my life would be over* [ID33; hypertensive].

Study participants were generally accepting of their diagnoses. Those who had experienced unexplained symptoms were grateful to have an explanation for this and access to treatment. Several noted that they had been diagnosed in the past and not ‘*accepted*’ or ‘*followed up’* on their diagnosis, but re-diagnosis following community screening had enabled active engagement in self-management and treatment. For some, this was specifically related to information provided by CHV and referral to primary care, as described below. Study participants recognised the importance of patient counselling by community health workers to support acceptance of diagnosis and treatment:

*As they screen people, they can attend to them…if they go somewhere where these services have not been introduced, those people don’t understand what diabetes is, they have not understood what hypertension is, unless they screen and tell them. They should also be counselled so they can accept they have these conditions, and then start taking medications.* [ID21, hypertensive and diabetic]

The role of counselling provided by CHV was seen to be especially important for people who do not experience symptoms:

*It is about explaining to them politely so that they may understand their condition, because there are some people who refuse medicines because they don’t believe, because they don’t feel any pain’* [ID41; hypertensive]

### Referral and follow-up

Participants described their appreciation of simple, specific procedures for referral to primary and secondary level services. Clear instructions, for example being given a specific date and location to attend hospital, helped patients to follow up and receive care. Some were also encouraged by community health workers, emphasising the importance and urgency of seeking care, for example: ‘*She told me not to delay in going to the hospital*, *[as] I had been found with hypertension and it was elevated’* [ID04; hypertensive].

Patients were instructed to attend regular NCD clinics so that healthcare providers could monitor their condition(s) and prescribe medication. Interview participants reported that they endeavoured to attend regularly, and some explained that they found information provided during their monitoring visits useful to track their progress in managing their condition. Three measurements were highlighted by patients; blood sugar level (diabetes), blood pressure (hypertension), and weight (for those who had been advised to lose weight). For asymptomatic patients, monitoring visits provided knowledge about their health status which patients did not otherwise have access to:

*If you are at home your pressure can rise and you don’t know because you don’t have the measuring machine. When you come here you are measured and then they tell you your state, that helps us* [ID17; hypertensive]

Making services available at lower level health facilities was widely seen to encourage regular clinic attendance, as was being given a choice of clinic, because it minimised travel and associated costs for some participants who now were able to access services closer to their home:

*I’m happy because it is close. [Location] was very far. I’m happy because I come here at 7am while in [location] we used to go at 5am….I’m happy here. I even walk from home to this place.* [ID08; diabetic]

However, those living further away from health facilities continued to experience major challenges to attend monitoring appointments regularly, in particular those with very limited financial resources; consultation fees charged by level 4 and 5 facilities also continued to pose a major barrier to access services for some participants.

Similarly, clinic wait times were experienced as challenging, with reports ranging from 4–6 hours to entire days, due to the volume of patients waiting. Long waits were especially problematic where they interfered with income-generating activities, with participants saying that they would delay or miss follow-up visits altogether. For frail people and those with diabetes, who had to attend clinics fasting, long waits frequently caused them to feel unwell: ‘*we will take long…*.*we always come when we have not eaten anything*. *When it reaches 1 or 2 pm you start to feel dizzy’* [ID10; diabetic and hypertensive].

While long waits could cause people to drop out of care, positive interactions with healthcare providers tended to support retention in care. Participants generally felt well treated by health care providers, which they identified as a strength of the care they received: ‘*you’re given a warm welcome and you’re not laughed at*. *It feels nice*’ [ID10; diabetic and hypertensive]. Participants gave examples of staff listening and responding to challenges they encountered in condition management, for example changing medication prescriptions in response to side effects and adapting dietary advice where first line alternatives were not available or affordable to patients. Participants also described receiving individually tailored advice, which enabled them to incorporate exercise into their daily routine, for example while travelling to work, doing farm work or playing with grandchildren, depending on the individual’s circumstances. Respondents reported that they themselves understood the advice they were given, but they commented that some older patients who spoke languages other than Kiswahili might need additional support:

*There are those who don’t understand, like the really old women who don’t understand. They only understand their language. Many a times they have problems*. [ID18; hypertensive]

In these cases, fellow patients sometimes helped to translate.

### Treatment and self-management support

PIC4C integrated services aimed to provide treatment and support patient’s management of their condition(s) through education and ongoing support. Interview participants showed clear understanding of strategies to self-manage their condition through diet and exercise, and of the importance of medication adherence. However, incorporating these into their daily lives was often seen to be challenging because of associated costs and variable availability of medicines. We explore each factor in turn.

#### Diet

Participants reported high levels of awareness of dietary self-management strategies for hypertension and diabetes, and of the importance to follow advice received at diagnosis and regular clinic visits. While most said that they had introduced some changes into their diets, they found the process of change challenging. Changing diet was more likely where participants experienced clear direct positive effects, for example *‘ever since I started following up with the meals*, *I’ve seen improvements in my body and I’m doing better’* [ID09; diabetic]. At the same time, many of the recommended foods, such as whole grains, vegetables, fruits, white meats and liquid cooking oil, were reported to be more expensive than people’s regular diets and cost was frequently described as a barrier to adhering consistently to dietary advice. For example, liquid cooking oil, a recommended substitute for solid cooking oil, was more costly and therefore ‘*really difficult’* or impossible to access for some participants. As well as being more expensive, recommended foods such as brown flour and brown rice were unavailable locally in some areas. Those with caring responsibilities additionally reported tensions between spending on more expensive foods for their own diet while also providing for dependents:

*Sometimes it’s a problem because you lack money to buy the millet* [cereal grain] *…. when you see the kids have to eat, you won’t buy the millet and leave the kids.* [ID31; diabetic]

People with diabetes gave examples of the direct health impacts they experienced when they lacked resources enabling them to follow dietary guidance:

*Say you are supposed to take millet ugali* [staple corn-flour based food] *and vegetables, and you lack, you’ll be forced to eat the normal one. And if you proceed that way, if your sugars had stabilised, you find they’ll shoot up again* [1D24; diabetic and hypertensive].

Participant’s accounts of efforts to manage their diets indicate that PIC4C services effectively provided knowledge and support, but people’s capacity to implement and sustain change was limited, largely, by their financial resources.

#### Exercise

Exercise did not involve a financial cost to patients as such and was widely reported as manageable by both people with hypertension and diabetes. Participants described receiving specific advice about exercise, tailored to their age and health status, which prompted them to incorporate a range of activities into their weekly or daily routines. These included farm work, cycling, walking longer distances, and skipping. Some described observing clear impacts of exercise on their health:

W*hen my blood sugar was 21, I came and did physical exercises as the doctors had explained. I went back and was tested; it was 14. I tested again and it reached 7.5…* [ID23; diabetic and hypertensive]

The main reported barrier to exercising was feeling too unwell to do so.

#### Medication

As noted, study participants understood the importance of medication adherence to manage their condition(s), which was explained to them and reinforced by health care providers at clinic visits. Some reported that they had initially ‘*not accepted’* the need to take daily medication, then changed their view when their condition deteriorated and affected their daily life, or they had been encouraged to try taking medication and found their condition improved. Many participants reported that medicine had helped to make them feel ‘*normal*’ and to ‘*recover*’ from debilitating symptoms, for example with regular treatment ‘*you will live a normal life just like the other person who doesn’t have hypertension’* [ID37; hypertensive].

The importance of daily medication was reinforced by negative changes experienced when participants did not have access to medication:

*When I lack medicine…I feel my body is weak like it is coming back, but when I get drugs, I go back to normal so I start looking for money to buy the medicine. So, I have to have medicine all the time.* [ID02; diabetic and hypertensive]

There was tension for many participants between having the knowledge that medication adherence was essential and being unable to consistently afford to purchase medicines. Affordability of medicines was widely reported as the main challenge to condition management: *‘I run out of drugs and when I want to come to refill*, *I lack finances…*.*the burden is on the part of the drugs*. *Paying for them* [ID38; hypertensive]. One participant noted that *‘there are many’* people in the community ‘*who started treatment for hypertension and stopped*’ [ID33; hypertensive]. Participants made a distinction between sometimes skipping or missing medication due to circumstances beyond their control, and stopping taking medication.

*I have tried to adhere strictly. I have never stopped. Maybe just lack money to go then I can skip* [ID29; diabetic]

Lower cost subsidised medication sold at PIC4C facilities helped people to afford medication, so supporting adherence. However, prescribed medications were not always available at the facility and patients then needed to travel elsewhere to purchase them at higher cost in private pharmacies:

*In paying for the medicine there is no challenge because when we go to AMPATH* [PIC4C] *the medicines are cheaper, not so expensive. But if you lack there* [medicines not available]*, you go outside* [and that] *is when you find it to be very expensive*. [ID35; hypertensive]

In rural areas, travelling to visit private pharmacies incurred additional travel costs. Unavailability of drugs and lack of financial resources to get them privately meant that patients needed to find ’work arounds’, for example by purchasing only small quantities.

As noted, one quarter of study participants (n = 11, 26%) reported health insurance (NHIF) coverage but even they experienced problems accessing medicine in public facilities free of charge:

*I had this card* [proof of NHIF insurance cover] *and I was paying for it but the surprising thing there is, you have paid* [insurance premium] *for this month but when you go to the hospital there are no drugs, they tell you to go and buy.* [ID24; diabetic and hypertensive]

Some people who had very limited financial resources found even subsidised medication costs difficult to afford, particularly those with both hypertension and diabetes, or who were prescribed several medications:

*Now, its two conditions, diabetes and hypertension, and it is a lot of money…[..]..sometimes I have no money* [ID27; diabetic and hypertensive]

As with diet-related costs, people with caring responsibilities noted that competing priorities for limited financial resources made it difficult to prioritise medication purchase.

Some of our participants had access to patient support groups, which were set up in some of the PIC4C sites, and which offered pooling of monthly financial contributions. These assisted some in obtaining medications (‘*When one gets sick they contribute for you…[…]…if I lack drugs*, *they contribute for me and I get the drugs* [ID01, diabetic and hypertensive]). These groups, where available and used, often provided significant social support, helped people learn about managing their condition and support medication adherence through peer education:

*There are these groups…[..]..there are people who have taken medicine for 20 or 30 years and for you maybe you just started the other day, so these ones can be your teacher….[…] another will say mine used to pain me when I started medicine but when I continued it [it improved]* [ID 28; diabetic]

### Change over time

Reflecting on whether or how things had changed between interviews (an interval of 8–10 months), people with hypertension and/or diabetes commonly highlighted the importance of ‘taking time’, in some cases significant periods of over a year, to adjust to and implement lifestyle changes and establish optimum medication regimes. Participants described instances of struggling to cope with the various demands that a diagnosis of chronic disease had placed on them and their families, such as being able to secure reliable sources of income to support their family and pay for medications. Patients cited the support from the treating doctor that had enabled them to cope with and address challenges to manage their condition, for example, difficulties to follow dietary advice, and how this had improved their situation over time:

*I have seen a lot of changes this year. I feel different not like before. I do my work the one I trained* [in]. *I continue with the drugs and I’m not as sick as I was last year.*

[Interviewer: You are saying that this year your health has improved?]

*My health has improved and I have lost weight. This is because I was obese. After I got advice from the doctors and I reduced and changed the food that I take, I have seen great change. I’m doing well.* [ID03, diabetic and hypertensive]

Additionally, we identified changes over time in some participants’ capacity to engage with services and adhere to treatment regimens, which was frequently shaped by their households’ vulnerability to financial shocks. One example includes unexpected illness of a family member, causing reprioritisation of household financial resources, which meant reduced clinic attendance and gaps in purchasing medication.

A key change at the system level that patients identified at their second interview was an increased volume of people accessing services (although this was not universally reported), for example:

*The blood pressure population has gone high. When you come to the dispensary the number of hypertensives is more* [ID15, hypertensive]

In some cases, this resulted in longer waiting times for patients unless staffing was expanded to meet the increased demand, although this was not widely observed, except in some cases (‘*the number [of health centre staff] increased and they became free to see us’*, meaning that ‘*right now you don’t stay for too long waiting’* [ID24; diabetic and hypertensive]). Participants also reported changes in clinic organisation, which separated NCD patients from general patients, and this was seen to reduce waiting times:

*Nowadays it has improved because we have our own room. When you arrive on clinic day, you just go to your room and you will be helped* [ID28; diabetic]

## Discussion

This study found that the PIC4C model of care was successful in enabling people with diabetes and/or hypertension to access screening services close to their homes and supported them to enter and sustain care. However, it proved challenging for the programme to deliver systematically on retaining people in care and help them adhere to their treatment/s in a consistent manner, in great part because of the wider system context within which PIC4C has had to operate. Specific constraints related to ensuring reliable and affordable access to medicines [18, 19], which could only partly be compensated for by the revolving fund pharmacy model implemented as part of PIC4C [20]. This meant that especially those with limited financial resources had to identify other ways of accessing medications, or forgo taking medicines altogether.

Limited health insurance (NHIF) coverage, variable content of coverage for those who subscribed, and limited availability of medications at public facilities meant that health insurance did not reliably support patients to access NCD medications. Within the wider implementation study, evaluation of the effectiveness of NHIF chronic illness cover in providing financial protection to households with hypertensive and diabetic patients found low depth of coverage among households with active NHIF subscription, with over 70% of out-of-pocket health care costs not covered [21]. An assessment of the responsiveness of the package to patients’ needs identified inadequate coverage of medicines and monitoring costs [22], findings corroborated in a study of financial protection provided by NHIF among rural informal workers in western Kenya [23].

Thus, despite improving overall access to care, lack of sufficient financial resources to pay for several aspects of treatment remained a key constraint and burden for patients using PIC4C integrated services. Lack of reliable access to medications is a known significant barrier to treatment adherence for chronic NCDs in resource constrained settings [24–27], and increasingly recognised as an important aspect of treatment burden [28].

We observed substantial variation in participants’ individual financial and personal circumstances which affected their capacity to effectively manage their condition, including adhering to medical treatment [12, 29]. Thus, people with caring responsibilities and those who did not have a source of income or financial support from family members experienced greater challenges adhering to treatment. These issues often intersected and were particularly acute among single women and older people without support networks. Changes in circumstances over time could also alter people’s capacity to engage with health services and treatment, as reported elsewhere in resource-constrained settings [30]. People with both diabetes and hypertension found it more challenging to adhere to treatment in a consistent manner, due to higher out-of-pocket costs for medicines and care and specific diabetic dietary requirements. Participants made distinctions between ‘skipping’ medication, or being unable to consistently obtain the foods needed to follow to dietary guidance, and ‘stopping’ medication or deliberately not following guidance, resonating with the concept of ‘unintentional non-adherence’, described as”largely driven by a lack of capacity or resources to take medications” [31, p. 1582].

Health care worker’s responsive approach to delivering care enabled some patients to work around resource constraints and reduce barriers to treatment adherence, for example through discussing challenges in accessing recommended foods and identifying appropriate substitutes. Individually tailored advice was also seen to be important in supporting patients to undertake exercise compatible with their life stage and daily routine. We further found that newly diagnosed patients took substantial time periods to adjust to and implement treatment, and that support from PIC4C staff through this process helped them to persevere and find a combination of lifestyle adjustments and medication to manage their condition. This resonates with social theory of ‘biographical disruption’ [32], which explores the fundamental challenges that diagnosis with a chronic condition can pose, requiring significant adjustments to daily life, which can take time to normalise. Overall, these findings point to the need to support health care workers to use flexible, contextually responsive approaches to effectively support patients’ self-efficacy in the face of economic and other constraints.

Perhaps counter-intuitively, some of the challenges in service delivery reported by patients were the consequence of improvements in timely diagnosis and linkage into care facilitated by PIC4C, leading to increased patient volumes. This highlights the need for intervention planners to carefully consider the likely knock-on effects and unintended consequences when designing new strategies that are likely to generate higher demand for services. In the case of PIC4C, this would include enhancing service capacity to support retention in care, including increasing the number of staff in monitoring clinics, and making available medication in the system to support effective condition management. This was addressed in some facilities through changes in clinic staffing or frequency and the introduction of revolving fund pharmacies, but these were not implemented everywhere.

### Strengths and limitations of study

Key strengths of our study were the training and experience of the field team, the in-depth nature of interviews conducted at two time points within a nested study design, which allowed analysis of temporal changes and use of qualitative and quantitative data. Drawing on different data sources particularly strengthened our understanding of the range and the impact of individual financial resources and affordability of services.

Our study participants were people who engaged with care, had participated in a survey and were willing to be interviewed for further research. We therefore did not include people who did not engage with the PIC4C model after diagnosis. We cannot be certain about the nature of bias this has introduced into our data but it is likely that our sample has lower presentation of people with poorer experiences of diagnosis and linkage into care, and/or those who may have experienced greater barriers to accessing care.

More importantly perhaps, we anticipated that there was a risk of social desirability bias, meaning participants reporting what they believe to be expected positive behaviours and a reluctance to voice concerns or be critical about the service and service providers. We aimed to address this possibility by explaining that there was a clear separation between the researcher/s undertaking the interview and health care workers involved in PIC4C service delivery. We also explained that the purpose of the study was to understand how services work for patients. Study tools, developed with input from senior and junior staff across the team were designed to include open-ended questions and probes for positive and negative experiences.

Our sample included people with diverse conditions who were recruited across two counties (total 22 facilities, 11 in each county). This enabled triangulation of interview and previously collected survey data to ensure a robust data set, and inclusion of demographic and income data.

The study was a partnership between Kenyan researchers with extensive experience in implementation and evaluation of interventions, and clinical experience of service delivery for chronic conditions in Western Kenya, and UK based researchers with experience of implementation research in care for chronic conditions in diverse settings. Regular debrief meetings were held during data collection. The field team was led by a Kenyan medical sociologist (VN) with extensive experience of collecting, analysing and reporting qualitative research. Data analysis was led by a UK based researcher (RW) with experience in primary data collection and translation, and in conducting qualitative analysis with international teams. This combination enabled both in-depth understanding of the context in which the data was generated, and analytical distance. Discussion of preliminary findings with stakeholders was valuable to discuss interpretation and highlight themes of policy interest for exploration in greater depth.

## Conclusion

We set out to examine the extent to which the PIC4C integrated service supported patients with hypertension and/or diabetes to overcome challenges of timely diagnosis, linkage and retention in care and adherence to treatment, from the perspective of those receiving care. Our study found that the PIC4C model of care successfully engaged people in accessing screening services, which enabled timely diagnosis and supported people to enter care. Retention in care and optimal treatment adherence were more challenging, due to the wider system context within which PIC4C operated and participants’ economic constraints. Despite these constraints, patients felt supported in adjusting lifestyle related factors when health care workers adopted flexible, contextually responsive approaches to help them work around financial barriers to dietary changes and embed exercise in their daily routines. The findings of this study are timely as the Kenyan government is currently rolling out community-based screening of hypertension and diabetes across the country and integrated care at primary level across 35 of the 47 counties nationally [1]. The roll-out would therefore benefit from the lessons documented here, and we would recommend strengthening the pharmaceutical supply systems as an important precondition.

## Supporting information

S1 Checklist Standards for reporting qualitative research (SRQR). (DOCX)

S2 Checklist Strengthening the reporting of observational studies in epidemiology (STROBE) guidelines for reporting observational studies. (DOCX)

S3 Checklist Inclusivity in global research. (DOCX)

S1 File Interview topic guides. (PDF)

S1 Table Non-participation in round 2 interviews. (DOCX)
