# Supplementary material for: Managing diabetes and hypertension in western Kenya: A qualitative study of experiences of patients supported by the primary health integrated care for chronic conditions (PIC4C) model of care
Source: PLOS Glob Public Health. 2024 Aug 15;4(8):e0003245. doi: 10.1371/journal.pgph.0003245 (PMC11326601; doi:10.1371/journal.pgph.0003245)
Supplement: S1 Checklist — (DOCX) [file pgph.0003245.s001.docx]

**Managing diabetes and hypertension in western Kenya: a qualitative study of experiences of patients supported by the Primary Health Integrated Care for Chronic Conditions (PIC4C) model of care : SRQR Checklist**^1^

| No. | Topic | Item | Page number |
| --- | --- | --- | --- |
|  | **Title and abstract** |  |  |
| S1 | Title | Concise description of the nature and topic of the study Identifying the study as qualitative or indicating the approach (e.g., ethnography, grounded  theory) or data collection methods (e.g., interview, focus group) is recommended | Page 1 |
| S2 | Abstract | Summary of key elements of the study using the abstract format of the intended publication; typically includes background, purpose, methods, results, and conclusions | Page 2 |
|  | **Introduction** |  |  |
| S3 | Problem formulation | Description and significance of the problem/phenomenon studied; review of relevant theory and empirical work; problem statement | Page 3 |
| S4 | Purpose or research question | Purpose of the study and specific objectives  or questions | Page 3, lines 67-71 |
|  | **Methods** |  |  |
| S5 | Qualitative approach and research paradigm | Qualitative approach (e.g., ethnography, grounded theory, case study, phenomenology, narrative research) and guiding theory if appropriate; identifying the research paradigm (e.g., postpositivist, constructivist/ interpretivist) is also recommended; rationale* | Page 4, lines 76-80  Page 5, lines 113-118 |
| S6 | Researcher characteristics and reflexivity | Researchers’ characteristics that may influence the research, including personal attributes, qualifications/experience, relationship with participants, assumptions, and/or presuppositions; potential or actual interaction between researchers’ characteristics and the research questions, approach, methods, results, and/or transferability. | Page 21 line 467 – Page 22 line 478-85, 489-499 |
| S7 | Context | Setting/site and salient contextual factors; rationale* | Page 4 lines 81-87  (Page 3 lines 57-65) |
| S8 | Sampling strategy | How and why research participants, documents, or events were selected; criteria for deciding when no further sampling was necessary, (e.g., sampling saturation); rationale* | Page 4, lines 89-99 |
| S9 | Ethical issues pertaining to human subjects | Documentation of approval by an appropriate ethics review board and participant consent, or explanation for lack thereof; other confidentiality and data security issues. | Page 6 lines 135-137  Page 5 lines 101-106, 121-123 |
| S10 | Data collection methods | Types of data collected; details of data collection procedures including (as appropriate) start and stop dates of data collection and analysis, iterative process, triangulation of sources/methods, and modification of procedures in response to evolving study findings; rationale. | Page 4 line 73-77, 96-99  Page 5 lines 109-118  Page 6: 129-133  Page 21 469-471 |
| S11 | Data collection instruments and technologies | Description of instruments (e.g., interview guides, questionnaires) and devices (e.g., audio recorders) used for data collection; if/how the instrument(s) changed over the course of the study | Page 5 -6 lines 112-129  Supplementary Information S2 Interview Topic Guides |
| S12 | Units of study | Number and relevant characteristics of participants, documents, or events included in the study; level of participation (could be reported in results). | Page 4 lines 94-99  Page 6-8 Lines 144- 153, Table 1 |
| S13 | Data processing | Methods for processing data prior to and during analysis, including transcription, data entry, data management and security, verification of data integrity, data coding, and anonymization/de- identification of excerpts. | Page 5-6 lines 120 -133  Page 21 lines 467-71  Page 22 lines 487- 488 |
| S14 | Data analysis | Process by which inferences, themes, etc., were identified and developed, including the researchers involved in data analysis; usually  references a specific paradigm or approach; rationale*. | Page 6 line 125-133  Page 22 lines 492-499  Page 8 lines 155-159 |
| S15 | Techniques to enhance trustworthiness | Techniques to enhance trustworthiness and credibility of data analysis (e.g., member checking, audit trail, triangulation); rationale.* | Page 5 lines 121-122  Page 6 lines 129-133  Page 21 lines 467-471, 478-488 |
|  | **Results/findings** |  |  |
| S16 | Synthesis and interpretation | Main findings (e.g., interpretations, inferences, and themes); might include development of a theory or model, or integration with prior research or theory. | Pages 8-18 |
| S17 | Links to empirical data | Evidence (e.g., quotes, field notes, text excerpts, photographs) to substantiate analytic findings. | Pages 7-8 (Table 1)  Pages 8-18 (quotes) |
|  | **Discussion** |  |  |
| S18 | Integration with prior work, implications, transferability and contributions  to the field | Short summary of main findings; explanation of how findings and  conclusions connect to, support, elaborate on, or challenge conclusions of  earlier scholarship; discussion of scope of application/generalizability;  identification of unique contribution(s) to scholarship in a discipline or field. | Pages 18 – 21 |
| S19 | Limitations | Trustworthiness and limitations of findings | Page 21 -22 line 472-92 |
|  | **Other** |  |  |
| S20 | Conflicts of interest | Potential sources of influence or perceived influence on  study conduct and conclusions; how these were managed | Conflicts of interest statement |
| S21 | Funding | Sources of funding and other support; role of funders in data collection, interpretation, and reporting. | Funding statement |

*The rationale should briefly discuss the justification for choosing that theory, approach, method or technique rather than other options available, the assumptions and limitations implicit in those choices, and how those choices influence study conclusions and transferability. As appropriate, the rationale for several items might be discussed together.

**Reference**

^1^ O'Brien BC, Harris IB, Beckman TJ, Reed DA, Cook DA. Standards for reporting qualitative research: a synthesis of recommendations. Acad Med. 2014;89(9):1245-1251.
