# Supplementary material for: Managing diabetes and hypertension in western Kenya: A qualitative study of experiences of patients supported by the primary health integrated care for chronic conditions (PIC4C) model of care
Source: PLOS Glob Public Health. 2024 Aug 15;4(8):e0003245. doi: 10.1371/journal.pgph.0003245 (PMC11326601; doi:10.1371/journal.pgph.0003245)
Supplement: S2 Checklist — (DOCX) [file pgph.0003245.s002.docx]

STROBE Statement—checklist of items that should be included in reports of observational studies

|  | Item No. | Recommendation | Page  No. | Relevant text from manuscript |
| --- | --- | --- | --- | --- |
| **Title and abstract** | 1 | (*a*) Indicate the study’s design with a commonly used term in the title or the abstract | 1,2 | ‘*a qualitative study’* (full title line 1, abstract line 27) |
|  |  | (*b*) Provide in the abstract an informative and balanced summary of what was done and what was found | 1 | lines 27-43 |
| Introduction | | | |  |
| Background/rationale | 2 | Explain the scientific background and rationale for the investigation being reported | 3 | lines 48-67 |
| Objectives | 3 | State specific objectives, including any prespecified hypotheses | 3 | lines 69-71 |
| Methods | | | |  |
| Study design | 4 | Present key elements of study design early in the paper | 4 | lines 73-78 |
| Setting | 5 | Describe the setting, locations, and relevant dates, including periods of recruitment, exposure, follow-up, and data collection | 4,5 | Setting and location: lines 80-85  Date reporting:  - Recruitment period line 99  - Data collection period lines 107-8, as appropriate for qualitative study reporting (SRQR Checklist item S10)  -Concept of ‘exposure’ not appropriate for this study design |
| Participants | 6 | (*a*) *Cohort study*—Give the eligibility criteria, and the sources and methods of selection of participants. Describe methods of follow-up  *Case-control study*—Give the eligibility criteria, and the sources and methods of case ascertainment and control selection. Give the rationale for the choice of cases and controls  *Cross-sectional study*—Give the eligibility criteria, and the sources and methods of selection of participants | 4 | This qualitative study is nested within a wider survey. Eligibility criteria and sampling strategy for the wider survey are reported in detail in reference (7).  Lines 89-90 explain eligibility criteria ‘*screened and hypertension and/or diabetes confirmed through PIC4C service efforts between 2018 and 2020’*. Lines 91-97 describe the purposive sampling strategy (SRQR Checklist item S8), as appropriate for the qualitative study design. |
|  |  | (*b*) *Cohort study*—For matched studies, give matching criteria and number of exposed and unexposed  *Case-control study*—For matched studies, give matching criteria and the number of controls per case |  | Not applicable to this study design |
| Variables | 7 | Clearly define all outcomes, exposures, predictors, potential confounders, and effect modifiers. Give diagnostic criteria, if applicable |  | Not applicable to this study design |
| Data sources/ measurement | 8* | For each variable of interest, give sources of data and details of methods of assessment (measurement). Describe comparability of assessment methods if there is more than one group |  | Not applicable to this study design |
| Bias | 9 | Describe any efforts to address potential sources of bias |  | Purposive sampling strategy: line 91-5  Strengths and limitations of study: lines 465-478 |
| Study size | 10 | Explain how the study size was arrived at | 4 | Line 91-97 |

Continued on next page

| Quantitative variables | 11 | Explain how quantitative variables were handled in the analyses. If applicable, describe which groupings were chosen and why | 6 | Line 123 *‘Demographic characteristics of the sample were described using existing survey data* |
| --- | --- | --- | --- | --- |
| Statistical methods | 12 | (*a*) Describe all statistical methods, including those used to control for confounding |  | Not applicable to this study design |
|  |  | (*b*) Describe any methods used to examine subgroups and interactions |  | Not applicable to this study design |
|  |  | (*c*) Explain how missing data were addressed |  | Not applicable to this study design |
|  |  | (*d*) *Cohort study*—If applicable, explain how loss to follow-up was addressed  *Case-control study*—If applicable, explain how matching of cases and controls was addressed  *Cross-sectional study*—If applicable, describe analytical methods taking account of sampling strategy |  |  |
|  |  | (*e*) Describe any sensitivity analyses |  | Not applicable to this study design |
| Results | | | | |
| Participants | 13* | (a) Report numbers of individuals at each stage of study—eg numbers potentially eligible, examined for eligibility, confirmed eligible, included in the study, completing follow-up, and analysed | 4,6 | Line 88 (wider survey), lines 138-139 (qualitative study reported here) |
|  |  | (b) Give reasons for non-participation at each stage |  | Supporting Information: Non-participation in Round 2 Interviews |
|  |  | (c) Consider use of a flow diagram |  | A flow diagram is not optimal for this qualitative study design which collected data at 2 points in time; non participation in second interview round is more informatively explained using a table (Supporting Information: Non participation in Round 2 Interview) |
| Descriptive data | 14* | (a) Give characteristics of study participants (eg demographic, clinical, social) and information on exposures and potential confounders | 6-8 | Lines 137-147 including table |
|  |  | (b) Indicate number of participants with missing data for each variable of interest | 7-8 | Indicated in Table 1 (n=0-3) |
|  |  | (c) *Cohort study*—Summarise follow-up time (eg, average and total amount) | 5 | Time periods are indicated in lines 107-108; as this is not an epidemiological study exact follow up time is not calculated |
| Outcome data | 15* | *Cohort study*—Report numbers of outcome events or summary measures over time |  | Not applicable to this study design |
|  |  | *Case-control study—*Report numbers in each exposure category, or summary measures of exposure |  | Not applicable to this study design |
|  |  | *Cross-sectional study—*Report numbers of outcome events or summary measures |  | Not applicable to this study design |
| Main results | 16 | (*a*) Give unadjusted estimates and, if applicable, confounder-adjusted estimates and their precision (eg, 95% confidence interval). Make clear which confounders were adjusted for and why they were included |  | Not applicable to this study design |
|  |  | (*b*) Report category boundaries when continuous variables were categorized |  | Not applicable to this study design |
|  |  | (*c*) If relevant, consider translating estimates of relative risk into absolute risk for a meaningful time period |  | Not applicable to this study design |

Continued on next page

| Other analyses | 17 | Report other analyses done—eg analyses of subgroups and interactions, and sensitivity analyses |  | Not applicable to this study design |
| --- | --- | --- | --- | --- |
| Discussion | | | | |
| Key results | 18 | Summarise key results with reference to study objectives | 16 | Lines 401-409 |
| Limitations | 19 | Discuss limitations of the study, taking into account sources of potential bias or imprecision. Discuss both direction and magnitude of any potential bias | 21-22 | Lines 459-492 |
| Interpretation | 20 | Give a cautious overall interpretation of results considering objectives, limitations, multiplicity of analyses, results from similar studies, and other relevant evidence | 19-21 | Lines 410-458 |
| Generalisability | 21 | Discuss the generalisability (external validity) of the study results | 22-23 | Lines 503-507 9 as appropriate to study design |
| Other information | |  | | |
| Funding | 22 | Give the source of funding and the role of the funders for the present study and, if applicable, for the original study on which the present article is based |  | Included in funding statement |

*Give information separately for cases and controls in case-control studies and, if applicable, for exposed and unexposed groups in cohort and cross-sectional studies.

**Note:** An Explanation and Elaboration article discusses each checklist item and gives methodological background and published examples of transparent reporting. The STROBE checklist is best used in conjunction with this article (freely available on the Web sites of PLoS Medicine at http://www.plosmedicine.org/, Annals of Internal Medicine at http://www.annals.org/, and Epidemiology at http://www.epidem.com/). Information on the STROBE Initiative is available at www.strobe-statement.org.
