# Supplementary material for: Managing diabetes and hypertension in western Kenya: A qualitative study of experiences of patients supported by the primary health integrated care for chronic conditions (PIC4C) model of care
Source: PLOS Glob Public Health. 2024 Aug 15;4(8):e0003245. doi: 10.1371/journal.pgph.0003245 (PMC11326601; doi:10.1371/journal.pgph.0003245)
Supplement: S1 File — (PDF) [file pgph.0003245.s004.pdf]

Interview Topic Guides

This document contains two Interview Topic Guides:

Patient Interview Topic Guide Round 1

Patient Interview Topic Guide Round 2

[Note: Institution logos removed for publication under CC-BY 4.0 licence]

[Institution logos removed]

Scaling up the primary health integrated care project for chronic conditions in Kenya: an implementation research project

Qualitative interview topic guide: For IDIs with Patients Round 1

IN-DEPTH INTERVIEW ID		
INTERVIEW DATE (DD/MM/YYYY)		[___|___/___|___/___|___]
INTERVIEW RESULT	01=completed<br>02=partially completed	
INTERVIEWER'S NAME:		

Instructions

First steps:
1. Please introduce yourself to the participant and ask them to introduce themselves
*2. Please ensure that **the participant has read (or has had read to them) the study information sheet**, outlining the aims of the study, how their data will be used, and potential risks and benefits of participating, and **has signed the informed consent form** for participation.*
3. Please confirm that the participant is happy to have their interview audio-recorded and that they know they can stop the interview at any point or refuse to answer any of the questions.

If the above steps are completed, you may proceed with the interview.

The questions do not need to be asked in the given order. You may decide which questions to ask and in what order according to the circumstances of the particular person. The idea is to allow respondents to talk about their experience following their own logic and to feel more relaxed with the interview process. You could use the probes provided to lead the conversation in a particular direction, or to remind them of things they have said earlier in the conversation. Do not directly read probes to the respondent after posing a questions, first give them time to answer the question in their own words. Encourage participants to provide concrete examples of issues they raise in their answers.

Please fill out the following (from PETS response):

Sex: ____
Age: ____
County: ____
Sub-County: ____
Facility name: ____
Reported health condition/s: ____

Introduction

1. Please tell me a little about your diabetes and/or hypertension. How long have you had this/these health problem/s?

2. When did you first notice that something was wrong and what did you do?
   Note: it might have been detected through screening, in which case ask for description of that experience
   Probe:
   - How, where and when was diabetes/hypertension diagnosed?
     - *What did you know about diabetes/hypertension before diagnosis?*
     - *How did you feel when your diabetes/hypertension was diagnosed?*
   - What has happened since you were diagnosed in terms of treatment?

Managing your health condition/s

3. Please tell me about your experiences of managing your health condition/s
   - Where do you look for information or advice on how to manage your diabetes/hypertension. How useful has this been?

4. What do you find difficult in managing your diabetes/hypertension and why?
   Probe for the following areas that they might find difficult and explore (a) 'Is there anything that you find especially difficult' and (b) 'Is there anything that you have found that makes it easier / does anyone help you with this':
   - Managing medications, e.g. organising or storing medicines; having to take more than one at regular intervals; need to adjust daily activities to align with medication schedule; side effects
   - Making and keeping medical appointments, e.g. finding time to get to the appointment; organising transport; having the energy
   - Monitoring health condition, e.g. checking blood sugar levels
   - Keeping to a particular diet/exercise regimen
   - Paying for medicines/medical appointments/healthy foods
   - Any other? E.g. any other health condition they might have to manage

5. How have the changes you have had to make to manage your diabetes/ hypertension affected your relationships with your family or friends?
   Probe:
   - Has it been a positive experience, and if so, in what way? e.g. family/friends are very supportive/help with management of condition
   - Has it created challenges, and if so, how? e.g. feeling dependent on others; family/friends do not understand the health problem

6. Please tell me about the kinds of support you are receiving from the wider community to help manage your health situation.
   Examples may include the local church, community support groups, etc.

Your experiences of health care for diabetes/hypertension

7. Please tell me about the kinds of care and support you have received at your last medical appointment for your diabetes/hypertension.
   Probe:
   - *What was the appointment for? e.g. follow-up, receiving a diagnostic test, etc.*
   - *What happened when you arrived at the facility/clinic? Whom did you see first (nurse, doctor, other)? And what happened then?*
   - *How has the care and support you received made you feel?*

8. Can you tell me about your general experience of medical appointments since you have been diagnosed with diabetes/hypertension?
   Probe:
   - *Who initiates medical appointments, the health facility or yourself? How is this arranged?*
   - *Are you able to see someone when you need to? Can you describe this to me?*
   - *How easy do you find it to attend medical appointments? How so?*

9. Have you experienced any challenges with keeping your appointments?
   Probe:
   - *If you have had to miss one or several medical appointments, can you tell me the reasons why you could not attend? E.g. cost, transportation, time away from work, other?*
   - *What happened then? E.g. did the health facility get in touch to make another appointment?*

10. Thinking about the care and services you have been receiving for your diabetes/ hypertension so far: How well do you feel listened to? Do you feel your problems are taken seriously?
    Probe:
    - *If yes, can you share an example of why you feel this way?*
    - *If no, can you share an example of why you feel this way?*
    - *If you think you are not listened to, do you feel able to talk to someone in the health facility (nurse/doctor/other) about this?*

11. Do you feel that the health service/treatment you are receiving helps you manage your diabetes/hypertension?

Probe:

- *If yes, please describe in what way. Examples may include*
    - *The doctor/nurse:*
        - *explains things in a way that you can understand it*
        - *discusses any problems you might have*
        - *provides you with a treatment plan so you know what you need to do*
        - *offers to telephone/email them if you need to ask more questions*
        - *makes you feel confident that you can manage your condition*
    - *Medicines / equipment:*
        - *Medicines are always available/affordable*
        - *Equipment is available and functioning*
        - *Other?*
- *If not, please describe why you feel this way.*
- *What would you find helpful?*

12. How well do you feel that the different people you are seeing for your health care work together?

    Probe:

    - *Do you have one main point of contact who knows about you and your condition/s and care/treatment plan? Can you give an example?*
    - *Do you have to explain your health problem each time you see a health care provider? How do you feel about that?*
    - *Do you feel that you are kept informed about the next steps in your care? If so, how so?*

13. How would you describe the information you have been given? Do you feel you have had sufficient say in the care you receive?

    Probe (note that this question relates to ongoing information about the managing the diabetes/hypertension):

    - *Does the information you are given cover what you like to know?*
    - *How easy do you find it to understand the information that you are given, do you need help (e.g. from a family member) to understand it?*
    - *How confident do you feel to make decisions about your care or to ask for more information or support?*
    - *How well do you think does the doctor/nurse take account of your personal circumstances/preferences when discussing your care? Can you share any examples of when this happened (or did not happen)?*

14. What do you think the doctor/nurse/facility that you typically visit could do differently to better help you manage your diabetes/hypertension?

Living with diabetes/hypertension

15. How do you generally feel about living with a chronic condition such as diabetes and/or /hypertension?

    Probe:

    - *How much does managing your diabetes/hypertension interfere with your daily life (work, family, leisure activities, etc)? Does this bother you?*
    - *Are there times when you feel angry or frustrated? Can you give an example of when this is likely to happen?*
    - *Do you sometimes think that you often have to rely on others? If so, in what way? How does this make you feel?*

16. How well do you think you are able to manage your condition overall?

    Probe:

    - *Are there times when you feel unable to monitor your condition or control your diet? How does this make you feel?*
    - *Have you had situations when you felt less able to cope and for example skipped a medical appointment or did not take your medication? Can you describe what happened?*

17. Is there anything else you feel is important to say about your experiences of living with diabetes/hypertension?

[Institution logos removed]

Scaling up the primary health integrated care project for chronic conditions in Kenya: an implementation research project

Qualitative interview topic guide: For IDIs with Patients Round 2

**IN-DEPTH INTERVIEW ID**		
**INTERVIEW DATE** (DD/MM/YYYY)		[___|___/___|___/___|___]
**INTERVIEW RESULT**	01=completed<br>02=partially completed	
**INTERVIEWER'S NAME:**		

<u>Instructions</u>

First steps:
1. Please introduce yourself to the participant and ask them to introduce themselves
2. Please ensure that ***the participant has read (or has had read to them) the study information sheet****, outlining the aims of the study, how their data will be used, and potential risks and benefits of participating, and* ***has signed the informed consent form*** *for participation.*
3. Please confirm that the participant consents to having their interview audio-recorded and that they know they can stop the interview at any point or refuse to answer any of the questions.

If the above steps are completed, you may proceed with the interview.

The questions do not need to be asked in the given order. You may decide which questions to ask and in what order according to the circumstances of the particular person. The idea is to allow respondents to talk about their experience following their own logic and to feel more relaxed with the interview process. You could use the probes provided to lead the conversation in a particular direction, or to remind them of things they have said earlier in the conversation. Do not directly read probes to the respondent after posing a question, first give them time to answer the question in their own words. Encourage participants to provide concrete examples of issues they raise in their answers.

Please fill out the following (from PETS response):

Sex: ____
Age: ____
County: ____
Sub-County: ____
Facility name: ____
Reported health condition/s: ____

Introduction

Last year [May/June/July] you spoke with my colleague about your diabetes and /or hypertension. I would like to learn more about your experiences with diabetes and/or hypertension since then, and particularly anything that has changed for you over that time.

1. Please could you remind me how long have you had this/these health condition/s?

Patient's experiences since first interview

2. How has your health been over the past year or so, when we spoke last? Has anything changed and if so, how?
   Interviewer: please encourage the patient to first talk in their own words, and use the probes if needed after that:
   - How has it been managing your health in that time? Describe anything to do with your condition(s) become that has become easier. Describe anything to do with your condition(s) become that has become more difficult?
   - How has it been coming to the health centre? Have you been able to come to the [health centre] regularly? (how so/why not/have there been any changes over past 12 months?)
   - How has it been accessing medication? Have you been able to access medication when you need it? (how so/why not/ have there been any changes over the past 12 months?)
   - Describe any other changes you have observed in your health? (e.g. diagnosis with another condition)

3. Describe any other changes/life events that have occurred in your life the last year?
   (For example things like changing or stopping a work activity, having a child come to live in the household)
   If yes:
   - How have these changes/events affected your health, or affected any of things you do to look after your health at all? If so, in what way(s)?

4. Tell me about how you manage your condition/s overall?
   Probe:
   - *Are there times when you feel unable to monitor your condition? How does this make you feel?*
   - *Are there times when you feel unable to do things like control your diet, exercise, to manage your condition? How does this make you feel?*
   - *Have you had situations when you felt less able to cope and for example skipped a medical appointment or did not take your medication? Can you describe what happened?*

5. Have the services available to you (here/at the facility you usually attend) stayed the same or changed?
   Interviewer: please choose relevant probe:
   - If they have stayed the same, how often have you come?
   - If they have changed, how so?
     - E.g. staff, medicines, equipment, cost, waiting time, organisation of clinics, numbers of patients

Patient's experiences in general:

When we spoke with you in [May/June/July], we also spoke with other patients from all over Busia and Trans-Nzoia, and they told us about their experiences.

6. Some people talked about needing to change their diet due to their condition. Is that something that you have needed to do, too?
   - what kind of information did you receive about food/drink, from where?
   - did you understand it, did you ask questions?
   - which changes did you find easier? Which changes were more difficult? If yes, who did you ask questions?
   - are there any situations where you have found it easier to apply changes? (probe for each change separately) Are there situations where you have found it more difficult? (probe for each change separately)
   - were you able to discuss any problems you had using healthier foods with your doctor, and how was that?

7. Some people talked about needing to manage stress/ emotional upset to help manage their condition. Is that something that you have experienced, too?
   - *if yes:*
     - can you describe a situation where this felt important?
     - does anyone else help you to do this? (E.g. family, community, health care provider))
     - are there any situations where it is easier?
     -are there any situations where it is more difficult?
     - do you feel you are able to discuss these issues with your doctor or nurse?
       - If so, how have they helped you?
       - If not, why do you think this is?

Looking ahead:

8. Considering your experiences of living with (various) health condition(s) so far: What would you say are the main things that you think will help support/help you in living with your health conditions?

9. Thinking about the health services you have used/received for your health conditions so far, which aspects of these do you see as most important for someone like you who has these health conditions?

   Probe if needed:
   - e.g. screening, monitoring at health centre, being given next appointment time, access to medicines
   - could also include outreach/mobile clinics

10. Again thinking about the health services you have used/received for your health condition(s), what do you think the doctors or nurses at the facility that you typically visit could do differently to better help you manage your condition(s)?

   Probe:
   - What could the facility do differently?

11. If health services for people with conditions such as yours were being set up in other part of Kenya in future, what advice would you give to:
   - the people/doctors/nurses planning and setting up the services?
   - patients using the services?

12. Aside from things that the health services can do to help you dealing better with your health problems, what other supports do you think would be really helpful to have and why?
   - *in your family or household?*
   - *in your community?*
   - *peer support?*

13. Is there anything else you feel is important to say about your experiences of living with this/these health condition(s)?
