# Supplementary material for: Managing diabetes and hypertension in western Kenya: A qualitative study of experiences of patients supported by the primary health integrated care for chronic conditions (PIC4C) model of care
Source: PLOS Glob Public Health. 2024 Aug 15;4(8):e0003245. doi: 10.1371/journal.pgph.0003245 (PMC11326601; doi:10.1371/journal.pgph.0003245)
Supplement: S1 Table — (DOCX) [file pgph.0003245.s005.docx]

Non-participation in Round 2 Interview

Two female and one males who participated in Round 1 interviews did not participate in Round 2 interviews. Reasons for non-participation are listed in the table below.

| **Reason for non-participation** | **Number (n)** |
| --- | --- |
| Not contactable | 1 |
| Travelling away from region during Round 2 data collection period | 1 |
| Admitted to hospital | 1 |
| **Total** | **3** |

**S1 Table: Reasons for non-participation in Round 2 Interview**
