# Supplementary material for: Managing diabetes and hypertension in western Kenya: A qualitative study of experiences of patients supported by the primary health integrated care for chronic conditions (PIC4C) model of care
Source: PLOS Glob Public Health. 2024 Aug 15;4(8):e0003245. doi: 10.1371/journal.pgph.0003245 (PMC11326601; doi:10.1371/journal.pgph.0003245)
Supplement: Attachment — Submitted filename: PGPH PIC4CSU Review response letter_ 220424.docx [file pgph.0003245.s007.docx]

22 April 2024

Dear Dr Draper,

We thank you and the anonymous peer reviewer for reviewing our manuscript titled ‘Managing diabetes and hypertension in western Kenya: a qualitative study of experiences of patients supported by the Primary Health Integrated Care for Chronic Conditions (PIC4C) model of care’ [PGPH-D-24-00127]. We appreciate the time and effort that was invested in providing constructive comments, which we believe have increased the quality of the manuscript.

In line with the comments and suggestions, we have:

1) Reviewed our reference list to ensure that it is complete and correct.
2) Uploaded a completed copy of PLOS’ questionnaire on inclusivity in global research as Supporting Information (S1), and referred to this in our manuscript.
3) Responded as detailed below, including uploading the completed STROBE checklist as Supporting Information (S5).
4) Revised the manuscript with revisions marked in tracked changes. Please note that updates to reference numbers are not marked as the software does not track these.

We hope that you will find our response and the changes we introduced satisfactory.

We look forward to hearing from you in due course.

Kind regards,

on behalf of all authors,

Dr Ruth Willis (corresponding author, joint first author)

Responses to Editor and Reviewer comments

| Editor/Reviewer suggestions  (Line numbers refer to original manuscript) | Authors’ response  (Line numbers refer to revised manuscript with changes marked) |
| --- | --- |
| Editors’ suggestion:  *‘In the abstract, the study is a described as a qualitative longitudinal study, which is a specific methodology that has unique characteristics and strengths as a qualitative approach. However, this was not mentioned or expounded upon in the methods. If the term ‘longitudinal’ was just intended to refer to interviews at 2 time points (but not necessarily to draw on the qualitative longitudinal research approach), then I would suggest just describing it as interviews at 2 time points, with the intention of capturing changes over time.’* | Thank you for the suggestion. We have revised the abstract as suggested. The revised text now reads:  “We conducted a qualitative study to explore the extent to which PIC4C integrated services supported people with hypertension and/or diabetes towards timely diagnosis and referral, treatment, follow-up and adherence, from the perspective of those receiving care. Semi-structured interviews were conducted with a purposively sampled patient cohort at two time points, with the intention of capturing changes over time” (p. 1, lines 27-32) |
| Reviewers’ Comments to the Author  *Pleasure ensure the paper includes all STROBE checklist requirements* | The STROBE checklist is designed to strengthen the reporting of observational studies in epidemiology. While our study is also observational, it uses qualitative methods, and we believe that the Standards for Reporting Qualitative Research (SRQR) checklist as specified in PLOS guidance via the EQUATOR Network is more appropriate. We included the SRQR checklist in our original submission but had not referenced it in the original manuscript. We have now rectified this oversight in the revised manuscript by adding a reference:  “We report study methods in accordance with the standards for reporting qualitative research (SRQR) guidelines (S1 Checklist) (7).”(p. 4, line 73-4).  We have additionally reviewed the paper according to the STROBE checklist and uploaded the completed checklist as Supporting Information (S5). Where checklist items are not relevant because of the qualitative study design we have noted this. |
| *There is a need for restructuring of the methods section of the manuscript.* | Thank you for this suggestion. We have restructured the text according to the specific suggestions as detailed below. |
| Line 72: *Separate headings for study design, this is the method of data collection and not the study design?* | We have added a separate heading ‘Study design’ (p. 4, line 75). |
| Line 75 re a larger quantitative survey *Is there a paper reference for this?* | Thank you for noting this. We have added the following reference to the quantitative study (no. 8; p. 4, line 79).  8. “Koros H, Nolte E, Kamano J, Mugo R, Murphy A, Naanyu V, et al. Understanding the treatment burden of people with chronic conditions in Kenya: A cross-sectional analysis using the Patient Experience with Treatment and Self-Management (PETS) questionnaire. PLOS Global Public Health. 2023;3(1):e0001407.” |
| Line 77-82 *This should be under a separate heading for setting and should describe the dates of recruitment and data collection* | As noted above, the reporting of this study follows the Standards for Reporting Qualitative Research (SRQR) checklist. However, you raise an important point and we have now changed the text to better signpost the different sections of the Methods as follows:   - We have added a separate heading ‘Study setting’ (p.4, line 81). This section describes contextual factors (SRQR Checklist item S7), and does not include details of recruitment and data collection. - We have added dates of recruitment for the qualitative study under ‘Sampling strategy and recruitment’ “(May – July 2021 and February – March 2022) “ (p. 4, line 101) (SRQR Checklist item S8) - We report dates of data collection for the qualitative study under ‘Data collection’ “24 May and 8 July 2021 (round 1) and between 24 February and 4 March 2022 (round 2)” (p. 4, lines 109-10) (SRQR Checklist item S10) |
| Line 84-86 *What was the criteria besides being willing, and exclusion criteria* | Thank you for noting this. Eligibility criteria and sampling strategy for the survey are reported in detail in reference (8). We briefly explain inclusion criteria further on p. 4, lines 91-93 where we note “Survey respondents had been screened and hypertension and/or diabetes confirmed through PIC4C service efforts between 2018 and 2020.”  In lines 93-99 (p. 4), we describe the purposive sampling strategy (SRQR Checklist item S8), as appropriate for the qualitative study design. |
| Line 86 *re survey methods to include above* | Reference to the the quantitative study added here as noted above (refence no. 8; p. 4, line 79). |
| Line 88 *How were these patients approached for the study?* | Our approach is explained on p. 5 (lines 101-104), in the ‘Sampling strategy and recruitment’ section, where we note:  “Potential participants were contacted by telephone (May – July 2021 and February – March 2022) by trained bilingual research assistants who explained the purpose of the interview and what participation would involve. For those agreeing to take part, an interview date most convenient for the participant was set.” |
| *Line 96 This is the recruitment process, and should be above in the heading.* | We agree and have now moved the relevant section (p. 5. line 100) |
| *Line 112 piloted with how many participants* | We have now clarified “piloted with four participants” (p. 5. line 119). |
| Some editing is required for grammatical errors and sentence structure. | Thank you for noting this, we have carefully reviewed the full manuscript and revised sections that were unclear. |
