# Supplementary material for: Managing diabetes and hypertension in western Kenya: A qualitative study of experiences of patients supported by the primary health integrated care for chronic conditions (PIC4C) model of care
Source: PLOS Glob Public Health. 2024 Aug 15;4(8):e0003245. doi: 10.1371/journal.pgph.0003245 (PMC11326601; doi:10.1371/journal.pgph.0003245)
Supplement: Peer review history

# Peer review: Decision Letter 0

**DOI:** 10.1371/journal.pgph.0003245.r001

**Authors:** Catherine Elizabeth Draper

3 Apr 2024

PGPH-D-24-00127

Managing diabetes and hypertension in western Kenya: a qualitative study of experiences of patients supported by the Primary Health Integrated Care for Chronic Conditions (PIC4C) model of care

PLOS Global Public Health

Dear Dr. Willis,

Thank you for submitting your manuscript to PLOS Global Public Health. After careful consideration, we feel that it has merit but does not fully meet PLOS Global Public Health’s publication criteria as it currently stands. Therefore, we invite you to submit a revised version of the manuscript that addresses the points raised during the review process.

==============================

I had significant challenges finding reviewers for your paper, and was only able to secure 1 review, which recommended minor revisions. Due to this recommendation, and to not further delay the review process, I have also reviewed the manuscript myself, and I concur with the reviewer's comments. This is a well-written and interesting study, and I appreciate the nuance captured in the qualitative data, and the authors' thoughtful reflections on the strengths and limitations of the study. I just have one further suggestion:

In the abstract, the study is a described as a qualitative longitudinal study, which is a specific methodology that has unique characteristics and strengths as a qualitative approach. However, this was not mentioned or expounded upon in the methods. If the term ‘longitudinal’ was just intended to refer to interviews at 2 time points (but not necessarily to draw on the qualitative longitudinal research approach), then I would suggest just describing it as interviews at 2 time points, with the intention of capturing changes over time.

==============================

Please submit your revised manuscript by May 03 2024 11:59PM. If you will need more time than this to complete your revisions, please reply to this message or contact the journal office at globalpubhealth@plos.org. When you're ready to submit your revision, log on to https://www.editorialmanager.com/pgph/ and select the 'Submissions Needing Revision' folder to locate your manuscript file.

Please include the following items when submitting your revised manuscript:

- A rebuttal letter that responds to each point raised by the editor and reviewer(s). You should upload this letter as a separate file labeled 'Response to Reviewers'.
- A marked-up copy of your manuscript that highlights changes made to the original version. You should upload this as a separate file labeled 'Revised Manuscript with Track Changes'.
- An unmarked version of your revised paper without tracked changes. You should upload this as a separate file labeled 'Manuscript'.

Guidelines for resubmitting your figure files are available below the reviewer comments at the end of this letter.

We look forward to receiving your revised manuscript.

Kind regards,

Catherine Elizabeth Draper

Academic Editor

PLOS Global Public Health

Journal Requirements:

1. Please review your reference list to ensure that it is complete and correct. If you have cited papers that have been retracted, please include the rationale for doing so in the manuscript text, or remove these references and replace them with relevant current references. Any changes to the reference list should be mentioned in the rebuttal letter that accompanies your revised manuscript. If you need to cite a retracted article, indicate the article’s retracted status in the References list and also include a citation and full reference for the retraction notice.

2. Please include a complete copy of PLOS’ questionnaire on inclusivity in global research in your revised manuscript. Our policy for research in this area aims to improve transparency in the reporting of research performed outside of researchers’ own country or community. The policy applies to researchers who have travelled to a different country to conduct research, research with Indigenous populations or their lands, and research on cultural artefacts. The questionnaire can also be requested at the journal’s discretion for any other submissions, even if these conditions are not met.  Please find more information on the policy and a link to download a blank copy of the questionnaire here: https://journals.plos.org/globalpublichealth/s/best-practices-in-research-reporting. Please upload a completed version of your questionnaire as Supporting Information when you resubmit your manuscript.

Reviewers' comments:

Reviewer's Responses to Questions

**Comments to the Author**

1. Does this manuscript meet PLOS Global Public Health’s publication criteria? Is the manuscript technically sound, and do the data support the conclusions? The manuscript must describe methodologically and ethically rigorous research with conclusions that are appropriately drawn based on the data presented.

Reviewer #1: Partly

**********

2. Has the statistical analysis been performed appropriately and rigorously?

Reviewer #1: N/A

**********

3. Have the authors made all data underlying the findings in their manuscript fully available (please refer to the Data Availability Statement at the start of the manuscript PDF file)?

The PLOS Data policy requires authors to make all data underlying the findings described in their manuscript fully available without restriction, with rare exception. The data should be provided as part of the manuscript or its supporting information, or deposited to a public repository. For example, in addition to summary statistics, the data points behind means, medians and variance measures should be available. If there are restrictions on publicly sharing data—e.g. participant privacy or use of data from a third party—those must be specified.

Reviewer #1: Yes

**********

4. Is the manuscript presented in an intelligible fashion and written in standard English?

PLOS Global Public Health does not copyedit accepted manuscripts, so the language in submitted articles must be clear, correct, and unambiguous. Any typographical or grammatical errors should be corrected at revision, so please note any specific errors here.

Reviewer #1: Yes

**********

5. Review Comments to the Author

Please use the space provided to explain your answers to the questions above. You may also include additional comments for the author, including concerns about dual publication, research ethics, or publication ethics. (Please upload your review as an attachment if it exceeds 20,000 characters)

Reviewer #1: Pleasure ensure the paper includes all STROBE checklist requirements, and see PDF attachment with comments for addressing.

There is a need for restructuring of the methods section of the manuscript. Some editing is required for grammatical errors and sentence structure.

**********

6. PLOS authors have the option to publish the peer review history of their article (what does this mean?). If published, this will include your full peer review and any attached files.

**Do you want your identity to be public for this peer review?** If you choose “no”, your identity will remain anonymous but your review may still be made public.

For information about this choice, including consent withdrawal, please see our Privacy Policy.

Reviewer #1: No

**********

[NOTE: If reviewer comments were submitted as an attachment file, they will be attached to this email and accessible via the submission site. Please log into your account, locate the manuscript record, and check for the action link "View Attachments". If this link does not appear, there are no attachment files.]

While revising your submission, please upload your figure files to the Preflight Analysis and Conversion Engine (PACE) digital diagnostic tool, https://pacev2.apexcovantage.com/. PACE helps ensure that figures meet PLOS requirements. To use PACE, you must first register as a user. Registration is free. Then, login and navigate to the UPLOAD tab, where you will find detailed instructions on how to use the tool. If you encounter any issues or have any questions when using PACE, please email PLOS at figures@plos.org. Please note that Supporting Information files do not need this step.

Attachment Submitted filename: PGPH-D-24-00127_RLC.pdf

# Author comment: Author response to Decision Letter 0

**DOI:** 10.1371/journal.pgph.0003245.r002

23 Apr 2024

Attachment Submitted filename: PGPH PIC4CSU Review response letter_ 220424.docx

# Editor report: Decision Letter 1

**DOI:** 10.1371/journal.pgph.0003245.r003

**Authors:** Catherine Elizabeth Draper

30 Apr 2024

Managing diabetes and hypertension in western Kenya: a qualitative study of experiences of patients supported by the Primary Health Integrated Care for Chronic Conditions (PIC4C) model of care

PGPH-D-24-00127R1

Dear Dr Willis,

We are pleased to inform you that your manuscript 'Managing diabetes and hypertension in western Kenya: a qualitative study of experiences of patients supported by the Primary Health Integrated Care for Chronic Conditions (PIC4C) model of care' has been provisionally accepted for publication in PLOS Global Public Health.

Before your manuscript can be formally accepted you will need to complete some formatting changes, which you will receive in a follow up email. A member of our team will be in touch with a set of requests.

Please note that your manuscript will not be scheduled for publication until you have made the required changes, so a swift response is appreciated.

IMPORTANT: The editorial review process is now complete. PLOS will only permit corrections to spelling, formatting or significant scientific errors from this point onwards. Requests for major changes, or any which affect the scientific understanding of your work, will cause delays to the publication date of your manuscript.

If your institution or institutions have a press office, please notify them about your upcoming paper to help maximize its impact. If they'll be preparing press materials, please inform our press team as soon as possible -- no later than 48 hours after receiving the formal acceptance. Your manuscript will remain under strict press embargo until 2 pm Eastern Time on the date of publication. For more information, please contact globalpubhealth@plos.org.

Thank you again for supporting Open Access publishing; we are looking forward to publishing your work in PLOS Global Public Health.

Best regards,

Catherine Elizabeth Draper

Academic Editor

PLOS Global Public Health

***********************************************************

Reviewer Comments (if any, and for reference):
